# Ligand-competent fractalkine receptor is expressed on exosomes

**DOI:** 10.1016/j.bbrep.2021.100932

**Published:** 2021-02-02

**Authors:** Eun Jeong Park, Phyoe Kyawe Myint, Michael G. Appiah, Patsorn Worawattananutai, Janjira Inprasit, Onmanee Prajuabjinda, Zay Yar Soe, Arong Gaowa, Eiji Kawamoto, Motomu Shimaoka

**Affiliations:** a Department of Molecular Pathobiology and Cell Adhesion Biology, Mie University Graduate School of Medicine, Tsu, Mie, 514-8507, Japan; b Department of Emergency and Disaster Medicine, Mie University Graduate School of Medicine, Tsu, Mie, 514-8507, Japan

**Keywords:** Exosome, Chemokine, Fractalkine, Chemokine receptor, CX3CR1

## Abstract

Expression of chemokine receptor CX3CR1 is reportedly restricted to several cell types including natural killer cells, cytotoxic T cells, monocytes, and macrophages. However, its expression and function on exosomes, which are nanosized extracellular vesicles known to act as mediators of intercellular communications, remain unclear. Here, we investigated CX3CR1 expression on exosomes isolated from various cell types. Although we found that all the exosomes tested in our study highly expressed CX3CR1, this chemokine receptor was expressed only inside, but barely on, their source cells. Moreover, exosomal CX3CR1 was capable of binding soluble CX3CL1. Therefore, our study suggests that CX3CR1 is a novel and ligand-competent exosome receptor.

## Highlights

• CX3CR1 is highly expressed by exosomes.
• Expression of CX3CR1 is restricted within, but not on, the cells.
• Exosomal CX3CR1 is capable of binding soluble CX3CL1.
• CX3CL1 binding of exosomes may deprive their source cells of the chance to bind this chemokine.

## Graphical abstract

**Figure:**
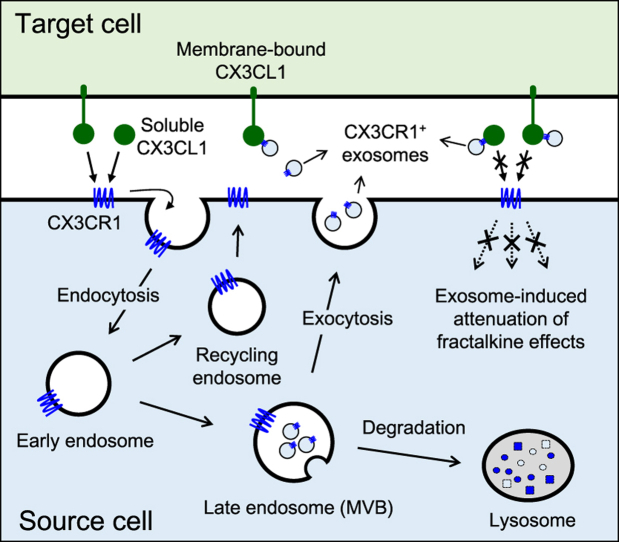

## 1 Introduction

Extracellular vesicles are cell-released lipid-bilayer particles and include exosomes, microvesicles, and apoptotic bodies that differ in size (40 nm–5 μm) [1]. Among them, exosomes are the nanosized vesicles (40 nm–150 nm) secreted after intracellular inbound sprouting of endosomes or multivesicular bodies (MVBs) [2]. The exosomes are effective at mediating cell-to-cell interaction by transporting bioactive materials [3]. Intercellular transport of exosomal mediators in a paracrine or autocrine fashion is thought to accelerate directional trafficking of cancer cells [4]. Functional components contained within exosomes may include integrins present on the surfaces and microRNAs (miRNAs) inside vesicles. Exosomal integrins serve as a molecular signature, particularly for organotropic metastasis; the integrins α6β4 (or α6β1) and αVβ5 of breast-cancer cell exosomes are necessary for lung- and liver-tropic metastases, respectively [5]. T-cell exosomes expressing integrin α4β7 negatively regulate the ligand expression in small intestines, presumably mediated by transferring functional miRNAs, and inhibit the subsequent homing of lymphocytes to the tissue [6]. Thus, exosomal integrins are involved in making pre-metastatic or pre-homing niches and influencing recruited cells.

Apart from their functionality vis-à-vis integrins, chemokine receptors on exosomes have been shown to play a role in tissue-specific homing or chemotaxis in both cells and exosomes. The CXCR4 and CCR2 on bone-marrow stromal cell-derived exosomes exert a promoting effect on the migration of multiple myeloma cells [7]. In addition, the splenic accumulation of dendritic cell (DC)-derived exosomes is mediated by CCR7 [8]. Exosomal chemokine receptors have been shown to be crucial to the exosome-induced amelioration of certain disease models. For example, mesenchymal stem cell-derived exosomes that highly express CCR2 mitigate both ischemia/reperfusion-induced renal injury and post-stroke cognitive impairment, via limiting CCL2-mediated macrophage activation [9,10]. In addition, the exosomes from mast cells are believed to alter immune responses by using CCR1 [11]. These reports show that multiple chemokine receptors play roles in determining the various functions of exosomes.

When cognate chemokines are bound, the chemokine receptors present on the plasma membrane are generally internalized in early endosomes and are then trafficked to MVBs [12]. These uptakes of chemokine receptors to inner organelles and consequent elimination on cell surface are indispensable physiological processes for restricting chemokine access [12,13]. Chemokine receptors in the MVBs are degraded upon their fusion to lysosomes or are recycled on the cell surface in order to refine receptor signaling after endosomal fusion to the plasma membrane [[14], [15], [16], [17]].

The chemokine receptor CX3CR1 mediates cell adhesion and trafficking through its interaction with chemokine CX3CL1 (fractalkine) [18]. CX3CR1 expression is detected by several cell types including natural killer cells, cytotoxic T cells, monocytes, and macrophages [[18], [19], [20]]. The binding of CX3CL1 to cellular CX3CR1 has been known to affect the pathogenesis underlying inflammations and cancers [[21], [22], [23], [24]]. In addition, the CX3CR1-CX3CL1 interaction has been found to be critical for DC migration in inflamed lymphatics [25]. Lymphatic endothelial cell-released exosomes have been shown to provide a lymphatic environment such that the directional migration of CX3CR1-expressing DCs is guided and augmented by inflammatory conditions [26]. Nonetheless, the exosomal expression and function of CX3CR1 remains to be elucidated.

Here, we examined the exosomal expression of CX3CR1 by using many cell types, revealing its high expression by all the exosomes used, and further showing the exosomal binding to CX3CL1. Based on the compelling results obtained in this study, CX3CR1 represents a ligand-competent chemokine receptor highly expressed by exosomes.

## 2 Materials and Methods

### 2.1 Cell culture

All the study has been done with mouse cell lines and primary cells, unless otherwise specified. The spleen cells isolated were used for *in vitro* culturing and activating primary T cells as previously described [6]. In brief, after eliminating erythrocytes by using ammonium-chloride-potassium (ACK) buffer (Thermo Fisher Scientific, Waltham, MA, USA), the lymphocytes were subjected to resuspension in RPMI1640 (Nacalai, Kyoto, Japan) containing 10% exosome-depleted fetal bovine serum (FBS) (Equitech-Bio, Kerrville, TX, USA) and penicillin/streptomycin (Nacalai). The cells were then plated on culture dishes coated with anti-CD3 (3 μg/ml) and anti-CD28 (3 μg/ml) antibodies (BD Biosciences, San Jose, CA, USA) in a 37 °C incubator with 5% CO_2_ supply. T cells activated for 48 h were moved onto antibody-uncoated dishes and further incubated for 72 h in the same culture media supplemented with interleukin-2 (1 ng/ml) (R&D Systems, Minneapolis, MN, USA). As mentioned above, to eliminate extracellular vesicles (EVs) including exosomes in FBS, FBS was centrifuged at 76,000 *g* for 18 h at 4 °C using polypropylene centrifuge tubes (Beckman Coulter, Brea, CA, USA), a swing bucket rotor (SW 28 Ti, Beckman Coulter) and an L60 Ultracentrifuge (Beckman Coulter) and the supernatant solution was filtered through 0.22-μm filter units (Merck, Darmstadt, Germany). This filtrated FBS was considered to be exosome-depleted and was used in this study for exosome isolation from the cells.

Several mouse tumor cell lines including TK1, CT26.WT, EL4, LTPA, and B16F10 cells were obtained from ATCC (Manassas, VA, USA). RAW264.7 (mouse macrophages) and THP-1 (human monocytes) were also from ATCC. MLO-Y4 (osteocyte) and MLO-A5 (osteoblast) cells were purchased from Kerafast (Boston, MA, USA). All cells were cultured, according to the manufacturers’ instructions, for 48 h in RPMI1640, DMEM (Nacalai), or MEMα (Thermo Fisher Scientific) media supplemented with 10% EV-depleted FBS and penicillin/streptomycin.

### 2.2 Mice

C57BL/6J mice (8–11 weeks old) were purchased from CLEA Japan (Tokyo, Japan) and maintained at the Experimental Animal Facility of Mie University. Experimental animal protocols were approved by the Ethics Review Committee for Animal Experimentation of Mie University (Approval number: #27-6-2). Spleens, bone marrows, and lungs were separated from the mice and used to isolate cells by using a mechanical dissociation with Falcon 40-μm cell strainers (Corning, Glendale, AZ, USA). The media collected in the cell isolation procedure were used to isolate exosomes (see section 2.3. for detail).

### 2.3 Isolation and characterization of exosomes

Exosomes were isolated as previously described with minor changes [6,27,28]. Briefly, culture media were centrifuged at 1000 *g* for 10 min at 4 °C to remove cells. The supernatant was spun at 2000 *g* for 20 min at 4 °C to eliminate apoptotic bodies. The supernatant was centrifuged in an L60 Ultracentrifuge (Beckman Coulter) at 24,000 *g* for 20 min at 4 °C. The supernatant was then subjected to a second centrifugation at 110,000 *g* for 2 h at 4 °C. The pelleted exosomes were subsequently suspended in phosphate-buffered saline (PBS) buffer (Nacalai). In turn, this exosome solution was passed through a 0.22-μm filter unit and spun at 110,000 *g* for 2 h at 4 °C. The pellet (exosomes) was suspended in PBS buffer. The concentration was measured with a bicinchoninic acid protein assay kit (Thermo Fisher Scientific). The particle size was characterized by using a dynamic light scattering (DLS) device (Horiba, Kyoto, Japan).

### 2.4 Exosome conjugation with microbeads

The exosomes were conjugated to 4-μm latex beads for efficient detection and then stained with fluorescently labeled monoclonal antibodies as previously shown [6]. In brief, after standardizing all different exosomes equally at 0.5 mg/ml in PBS, the exosomes (5 μg) were conjugated to microbeads (10 μl) (Thermo Fisher Scientific) in 1 ml of PBS by incubating the mixture for 2 h using a tube rotator and then blocked by an incubation with 100 mM glycine for 30 min. The exosomes were washed three times with PBS containing 0.5% bovine serum albumine (Sigma, St. Louis, MO, USA). The same amounts of exosome samples (5 μg) coupled to 10 μl latex beads were subjected to flow cytometry below so that the expressions are detected feasibly at comparable amounts of exosomes. In some experiments, 1 ml of PBS, 1 ml of EV-depleted FBS, or 1 ml of FBS were conjugated with 10 μl latex beads as done with the same methods as exosomes and then assessed for any expressions via using flow cytometry.

### 2.5 Flow cytometry analysis

Antibodies to CD9 (HI9a), CD63 (NVG-2), CD63 (H5C6), CD81 (Eat-2), CCR9 (9B1), CXCR4 (L276F12), and CX3CR1 (SA011F11) were purchased from BioLegend (San Diego, CA). Isotype controls including Rat IgG2a, Rat IgG2b, Armenian Hamster IgG, Mouse IgG2a, and Mouse IgG1 were also from BioLegend. The antibody to CD9 (KMC8) was obtained from BD Biosciences. The antibodies to CCR7 (4B12), CCR10 (248918) were acquired from R&D Systems. The cells or microbead-conjugated exosomes were stained with the fluorescently labeled antibodies, washed twice with PBS containing 2% FBS and 2 mM ethylenediaminetetraacetic acid (EDTA) (Wako, Osaka, Japan), and analyzed by using BD Accuri C6 flow cytometer and software (BD Biosciences). For this method of microbead conjugation of exosomes, only positive events can be detected and fluorescently quantified in the flow cytometry. In some experiments, total (intracellular plus surface) staining experiment was done by using FIX and PERM Kit (Thermo Fisher Scientific) according to manufacturer's instructions.

### 2.6 Binding assay

The exosomes conjugated to the microbeads were incubated with recombinant mouse fractalkine-His (R&D Systems) at 37 °C for 1 h, washed once with PBS containing 2% FBS and 2 mM EDTA, and labeled with phycoerythrin (PE)-anti-His Tag antibody (Biolegend). After wash, fluorescence intensity for fractalkine binding was analyzed by using BD Accuri C6 flow cytometer and software.

## 3 Results and discussion

### 3.1 T-cell exosomes exhibit marked expression of chemokine receptor CX3CR1

**Fig. 1:**
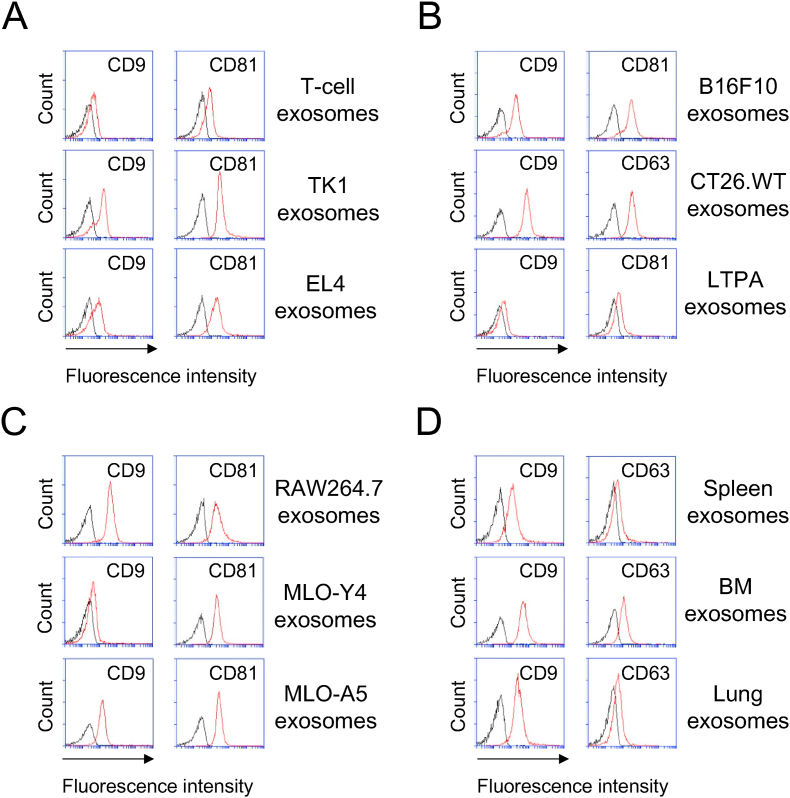
**Expression of tetraspanins on exosomes isolated from mouse cells.** The exosomes were isolated from the culture media, immobilized on microbeads, and stained with the indicated monoclonal antibody (MAb) (see Materials and Methods for details). The tetraspanin expressions were analyzed by using flow cytometry for the exosomes of mouse T cells or T-cell lines (**A**), mouse cancer cell lines (B16F10, CT26.WT, and LTPA) (**B**), mouse macrophage (RAW264.7) and bone (MLO-Y4 and MLO-A5) cell lines (**C**), and mouse primary cells isolated from spleens, bone marrows, and lungs (**D**). Flow cytometry histograms show the expression of the indicated markers. Data are representative of three separate analyses. Red lines, MAb; and black lines, isotype. (For interpretation of the references to colour in this figure legend, the reader is referred to the Web version of this article.)

To characterize the exosomes, we examined expression of tetraspanin markers (e.g., CD9, CD63, CD81, etc.). All the exosomes isolated from different types of mouse cells used in this study were positive for indicated tetraspanins, although they exhibited the expression levels different depending on source cells (Fig. 1A–D). We then examined the diameter, using DLS, of the exosomes including those which were isolated from TK1, EL4, B16F10, and CT26.WT cells. The exosomes from those cell types had similar and typical diameters (100–150 nm) (Supplemental Fig. 1). In our previous report, TK1 and T-cell exosomes were already validated for their particle number and size as well as CD9 and Alix expression by using nanoparticle tracking analysis (NTA) and Western blot analysis, respectively [6]. Taken together, the nanosized EVs (exosomes) used in current study were confirmed for their size and marker expression.

**Fig. 2:**
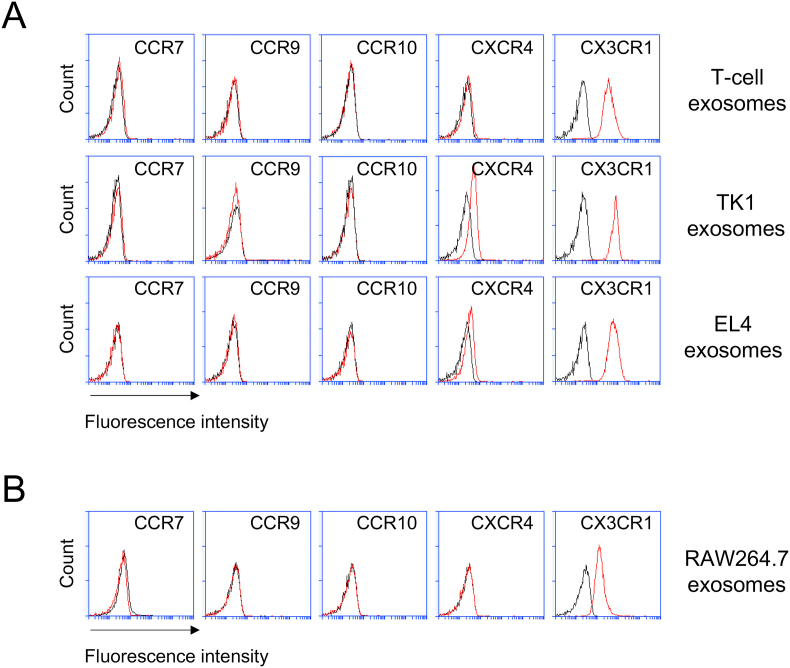
**Expression of selected chemokine receptors on mouse T-cell and two T-cell line exosomes and macrophage exosomes.** The exosomes were isolated from the culture media of T cells (activated T cells, TK1 cells, and EL4 cells) (**A**) and macrophage (RAW264.7) (**B**), immobilized on microbeads, and stained with the indicated monoclonal antibody (MAb) (see Materials and Methods for details). Exosomal expressions of chemokine receptors were examined by using flow cytometry analysis. CCR9, C–C chemokine receptor type 9; CXCR4, C-X-C chemokine receptor type 4; and CX3CR1, C-X3-C motif chemokin receptor 1. Flow cytometry histograms show the expression of the indicated chemokine receptors. Data are representative of three separate analyses. Red lines, MAb; and black lines, isotype. (For interpretation of the references to colour in this figure legend, the reader is referred to the Web version of this article.)

We sought to examine expression pattern of selected chemokine receptors on T-cell exosomes that were isolated from activated primary T cells [6]. We discovered that chemokine receptors including CCR7, CCR9, CCR10, and CXCR4 were barely expressed on the T-cell exosomes (Fig. 2A). However, the expression of chemokine receptor CX3CR1 proved remarkable on the exosomes (Fig. 2A). We next asked if these chemokine receptor expression patterns were evident on the exosomes isolated from T-cell lines such as TK1 and EL4 cells. While TK1 and EL4 exosomes exhibited slight expression of CXCR4 and little or no expressions of CCR9, CCR7, and CCR10, both exosomes also showed marked expression of CX3CR1, as did primary T cells (Fig. 2A). Together, the exosomes of mouse T cells and two T-cell lines, which were confirmed to be positive for CD9 and CD81 (Fig. 1A), expressed an extraordinary level of CX3CR1. We next examined these chemokine receptor expression patterns on exosomes of macrophage (RAW264.7) after validation of exosome marker expression (Fig. 1C). Moderate levels of CX3CR1, but no expressions of other receptors, were detected on RAW264.7 exosomes (Fig. 2B). In terms of CX3CR1 expression by the exosomes of immune cells such as T cells and macrophage, these results suggest that CX3CR1 is superior to other chemokine receptors.

### 3.2 Expression of CX3CR1 within, but not on, the cells reflects its preferential distribution on exosomes

**Fig. 3:**
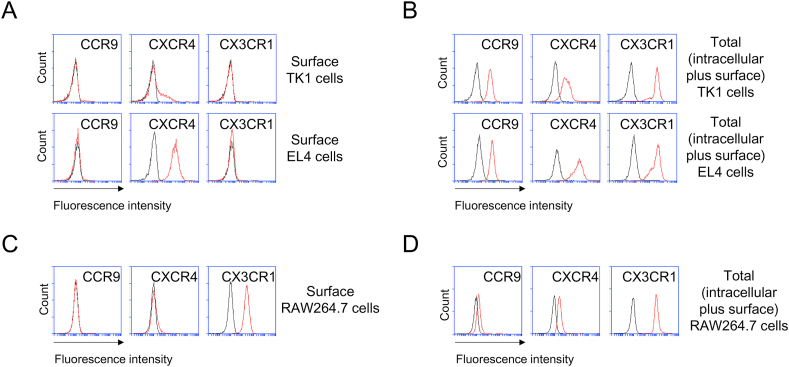
**Expression of CCR9, CXCR4, and CX3CR1 by TK1, EL4, and RAW264.7 cells.** Expressions of CCR9, CXCR4, or CX3CR1 on surface (**A**&**C**) or total (intracellular plus surface) compartments (**B**&**D**) of TK1 and EL4 (**A**&**B**), and RAW264.7 (**C**&**D**) cells were measured by using flow cytometry. CCR9, C–C chemokine receptor type 9; CXCR4, C-X-C chemokine receptor type 4; and CX3CR1, C-X3-C motif chemokin receptor 1. Flow cytometry histograms show the expression of the indicated chemokine receptors. Data are representative of three separate analyses. Red lines, MAb; and black lines, isotype. (For interpretation of the references to colour in this figure legend, the reader is referred to the Web version of this article.)

We next inquired whether CX3CR1 levels are intracellularly sustained, by using two T-cell lines (TK1 and EL4). Expressions of CCR9, CXCR4, and CX3CR1 were poor on the surface of TK1 and EL4 cells, except for CXCR4, which was moderately expressed by EL4 cells (Fig. 3A). In the case of CXCR4, both exosomes were able to acquire it at slight but comparable level, although only EL4 cells exhibited high CXCR4 expression in their outer and inner compartments (Fig. 2, Fig. 3B). Also, TK1 and EL4 cells appear to seldom share intracellular CCR9 with their exosomes, despite the fact that its intracellular expression was ample (Fig. 2, Fig. 3B). Both T cells contained considerable total levels of CCR9, CXCR4, and CX3CR1 (Fig. 3B), implying that CX3CR1-positive compartment is restricted to intracellular region. Conroy et al. have shown that soluble CX3CL1 engages in CX3CR1 internalization into human memory T cells and keeps this receptor level low on their surface [29]. However, because we didn't treat chemokine to activate cells in this study, no expression of CX3CR1 on surface of TK1 and EL4 cells (Fig. 3A) is unlikely due to chemokine-induced internalization.

Some of macrophages have been known to express CX3CR1 [30,31]. Using RAW264.7 cells, we sought to determine chemokine receptor expression on surface and in intracellular region. Intriguingly, substantial amounts of CX3CR1 were expressed on the surface (Fig. 3C) and much higher CX3CR1 expression was detected after permeabilization of RAW264.7 cells (Fig. 3D), suggesting a balanced CX3CR1 distribution in both compartments of RAW264.7. But, neither CCR9 nor CXCR4 was detected on the surface (Fig. 3C), and only a tiny degree of both receptors was contained in the intracellular region of RAW364.7 cells, compared to TK1 and EL4 cells (Fig. 3B&D).

CX3CR1 was superior to other chemokine receptors in its exosomal acquisition, suggesting the possibility that the intracellular compartment of this chemokine receptor might largely be restricted to the membrane of MVBs [[32], [33], [34]]. In this regard, the distribution of intracellular CX3CR1 appears to be biased toward the endosomal membranes of TK1 and EL4, instead of toward the plasma membranes. The CX3CR1 expression by exosomes and intracellular compartments was unequivocal compared to other chemokine receptors (CCR9 and CXCR4). Consequently, CX3CR1 represents a distinctive chemokine receptor preferentially expressed on the exosomes of immune cells such as T cells and macrophage. Indeed, chemokine receptors may harbor completely different characteristics depending on their relocation to the intracellular, membrane-bound extracellular, or endosomal compartments. Although the factors that determine the spatial relocation of different chemokine receptors remain unknown, only in CX3CR1 intracellular and exosomal expression levels synchronized. Unlike T cells, the macrophages (RAW264.7) expressed moderate levels of CX3CR1 on both cell surface and exosomes, possibly implying a reciprocal association between internalization and exosomal expression of this chemokine receptor. The spatially distinctive expression patterns of these three chemokine receptors of TK1, EL4, and RAW264.7 cells and exosomes are summarized in Table 1.

**Table 1:**
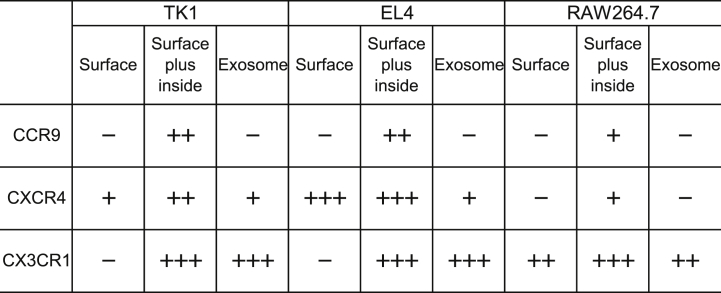
. Comparison of CCR9, CXCR4, and CX3CR1 for their spatially distinctive expressions in TK1, EL4, and RAW264.7 cells.

### 3.3 CX3CR1 represents a highly expressed chemokine receptor on exosomes

**Fig. 4:**
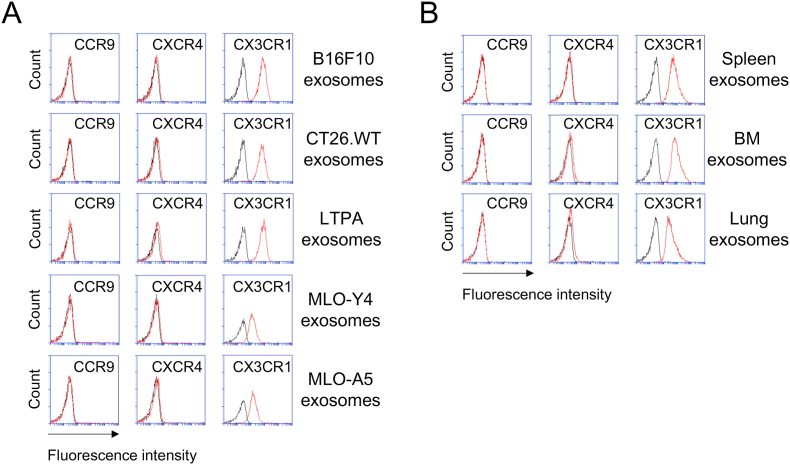
**Expression of CCR9, CXCR4, and CX3CR1 on the exosomes of various cell types.** The exosomes isolated from the culture media of various cell types after being cultured for 48 h were immobilized on microbeads and stained with the indicated monoclonal antibody (MAb) (see Materials and Methods for details). The expression levels of those chemokine receptors on the exosomes of several mouse cell lines (B16F10, CT26.WT, LTPA, MLO-Y4 and MLO-A5) (**A**) and mouse primary cells isolated from spleen, bone marrow, and lung (**B**) were measured by using flow cytometry. The exosomes were immobilized on microbeads, stained with the indicated MAb, subjected to flow cytometry and analyzed for the expression of those chemokine receptors. CCR9, C–C chemokine receptor type 9; CXCR4, C-X-C chemokine receptor type 4; and CX3CR1, C-X3-C motif chemokin receptor 1. Flow cytometry histograms show the expression of the indicated chemokine receptors. Data are representative of three separate analyses. Red lines, MAb; and black lines, isotype. (For interpretation of the references to colour in this figure legend, the reader is referred to the Web version of this article.)

To determine whether CX3CR1 expression by exosomes is limited to those isolated from the aforementioned cell types or is otherwise widespread, we used various cell lines to isolate their exosomes separately and examined their expression levels of CX3CR1, CCR9, and CXCR4. The exosomes from several cancer cells including B16F10 (mouse melanoma; epithelial-like cells), CT26.WT (mouse colon carcinoma; fibroblasts), and LTPA (mouse pancreatic adenocarcinoma; epithelial cells) were chosen to test their chemokine-receptor expression levels. These cancer-cell exosomes were found to express typical tetraspanins with some variations depending on their parent cell types (Fig. 1B). We found that all of the exosomes exhibited expression patterns that were similar to each other, in which high levels of CX3CR1 and no or only minute levels of CCR9 and CXCR4 were detected (Fig. 4A). Moreover, the exosomes isolated from two non-cancer bone cell lines, MLO-Y4 (mouse osteocytes) and MLO-A5 (mouse osteoblasts) (Fig. 1C), displayed a similar trend in expressing considerable levels of CX3CR1 and little levels of CCR9 and CXCR4 (Fig. 4A). Therefore, these results raise a possibility that CX3CR1 is a prominent chemokine receptor highly expressed by the exosomes isolated from all of the cell types examined in this study.

Next, we isolated the exosomes from spleen, bone marrow (BM), and lung cells of mice and then confirmed for their CD9 and CD63 expressions (Fig. 1D), in order to further validate the prevalent CX3CR1 expression on primary-cell exosomes which are presumably derived from multiple cellular sources. To this end, we assessed surface and total (intracellular plus surface) expression levels. The level of CX3CR1 was retained in the intracellular compartments of all three tissues to a high degree, while the levels on the cell surface proved extremely low compared to those inside the cells (data not shown), like all of the other cells tested. Intriguingly, their exosomes expressed a remarkable level of CX3CR1, while their expression levels of CCR9 and CXCR4 were negligible by comparison (Fig. 4B), suggesting that all of the exosomes tested in this study were intensely positive for CX3CR1.

In order to rule out a possibility that this marked CX3CR1 expression of the exosomes may be manifested due to any remaining EVs in their depleted FBS supplemented to culture media, we next decided to test this. PBS (1 ml), PBS (1 ml) plus EL4 EV sample (5 μg), EV-depleted FBS (1 ml), and FBS (1 ml) were first coupled to 10 μl latex beads as done with the same methods as exosomes. Then, all samples stained equally for CX3CR1 as well as CD9 and CD63 were analyzed using flow cytometry. On note, all three samples except for EL4 EV-containing conjugates did not show any positive signals for the expression of CD9, CD63, and CX3CR1 (Supplemental Fig. 2A). Therefore, these results suggest that expressions of CX3CR1 as well as tetraspanins are inherent to exosomes and unlikely affected by the possibly remained FBS-EVs in the culture media used in this study. Still, it will be worthy to carefully investigate the remaining EV levels in the FBS in near future [35,36].

Furthermore, because expression of CX3CR1 was remarkable on all the exosomes tested in current study, it may be open to question that the anti-CX3CR1 antibody is too blunt to discriminate the exosomes positive from negative for mouse CX3CR1. We thus tested this by using the exosomes isolated from THP-1 cells (human monocytes) as a negative control, despite CX3CR1 expression on human exosomes remains obscure and should be carefully determined in a separate experimental setting in near future. THP-1 exosomes positive for human CD9 and CD63 were negative for CX3CR1 (Supplemental Fig. 2B), indicating that the antibody to CX3CR1 used in this study was positive only to all tested mouse exosomes.

### 3.4 Exosomal CX3CR1 is capable of binding soluble CX3CL1

**Fig. 5:**
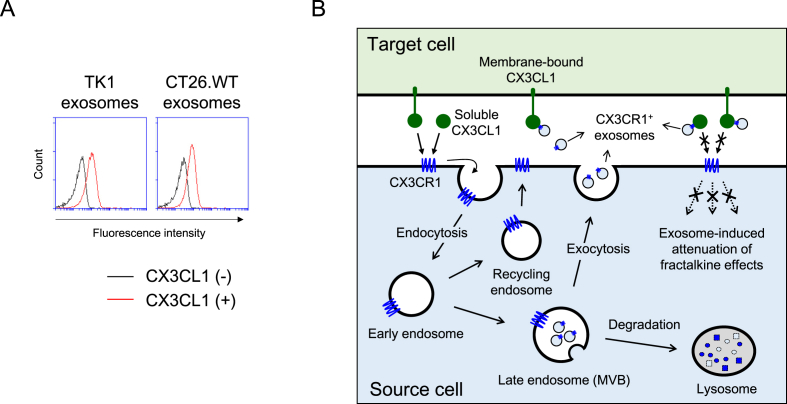
**Exosomal binding to CX3CL1 and a proposed model illustrating the effects of exosomal CX3CR1.** (**A**) The microbead-conjugated exosomes were incubated with or without soluble recombinant CX3CL1 that contained C-terminal six histidine residues and then stained anti-His Tag labeled with phycoerythrin. Exosomal binding was determined by using flow cytometry analysis. Data are representative of three separate analyses. (**B**) The binding of membrane-bound or soluble CX3CL1 to cellular CX3CR1 may lead to internalization of CX3CR1 in early endosomes and trafficking to MVBs, thereby downregulating its level on the cell surface. Intracellular CX3CR1 can be degraded upon MVB fusion to lysosomes or recycled to the plasma membrane. By contrast, MVBs undergo fusion to plasma membranes via exocytosis in order to release exosomes. As a compelling and convincing scenario, those exosomes highly expressing CX3CR1 bind CX3CL1 to regulate fractalkine-triggered signaling to their source cells. Thus, fractalkine binding by exosomes may prevent intercellular CX3CR1/CX3CL1 crosstalk. This hypothesis represents a novel model for exosomal downregulation of fractalkine's effects on source cells.

To examine the function of exosomal CX3CR1, we explored its binding to fractalkine (CX3CL1), a ligand unique to CX3CR1 [18]. We chose the exosomes isolated from TK1 and CT26.WT cells, which represent different lineages, lymphoid and myeloid, respectively, to ascertain their common activity despite being from distant sources. We conducted the binding assasy by using flow cytometry. The microbead-conjugated exosomes were incubated with recombinant fractalkine tagged by six histidine residues followed by fluorescently labeled anti-His Tag antibodies. The binding levels were measured by their fluorescence intensities. As shown in Fig. 5A, both exosomes exhibited an increase in their fluorescence intensities, which indicated binding to soluble fractalkine, possibly through exosomal CX3CR1. These results suggest that the exosomes from two different source cells were able to bind CX3CL1 through CX3CR1, possibly at comparable levels of adhesiveness.

Based on the results shown in this study, two possibilities regarding the regulatory function of CX3CR1-expressing exosomes can be raised. First, those exosomes expressing CX3CR1 are thought to bind both membrane-bound and soluble CX3CL1 produced by counterpart cells before do source cells. The exosomes isolated from the two selected cell types proved to be capable of binding recombinant CX3CL1 (Fig. 5A), thus supporting this hypothesis. Second, fractalkine binding of the exosomes may deprive their source cells of the chance to bind this chemokine, which may attenuate fractalkine-triggered pathophysiological effects. The proposed model illustrating the impact of exosomal CX3CR1 on fractalkine binding and intracellular pathways is shown in Fig. 5B.

Ours is the first study to report exosomal expression of chemokine receptor CX3CR1. In particular, CX3CR1 was prevalently and highly expressed by all of the exosomes isolated from the different cell types tested in this work. The various source cells contained high level of CX3CR1 in their intracellular compartments, presumably on the membrane of MVBs, which are the exosomal reservoirs inside cells. Moreover, those exosomes were capable of binding fractalkine. Our study concludes that CX3CR1 is a novel and ligand-competent molecule of exosomes. In future investigations, it would be worthwhile to elucidate: i) the underlying mechanisms by which CX3CR1 expression is maintained at such high levels on all of the exosomes; and ii) the exosomal impact on any functional changes undergone by the cells or tissues that express membrane-bound CX3CL1.

## Author statement

**Eun Jeong Park:** Conceptualization, Methodology, Validation, Formal analysis, Investigation, Writing - original draft, review & editing, Visualization, Funding acquisition. **Phyoe Kyawe Myint:** Investigation, Methodology. **Michael G. Appiah:** Investigation, Methodology. **Patsorn Worawattananutai:** Investigation, Methodology. **Janjira Inprasit**: Investigation, Methodology. **Onmanee Prajuabjinda:** Investigation, Methodology. **Zay Yar Soe:** Investigation, Methodology. **Arong Gaowa:** Investigation, Methodology. **Eiji Kawamoto:** Investigation, Methodology. **Motomu Shimaoka:** Conceptualization, Data curation, Supervision, Project administration, Funding acquisition.

## Declaration of competing interest

The authors declare that they have no known competing financial interests or personal relationships that might have influenced the work reported in this paper.

## References

[1] Raposo G., Stoorvogel W. (2013). Extracellular vesicles: exosomes, microvesicles, and friends. J. Cell Biol..

[2] Denzer K., Kleijmeer M.J., Heijnen H.F., Stoorvogel W., Geuze H.J. (2000). Exosome: from internal vesicle of the multivesicular body to intercellular signaling device. J. Cell Sci..

[3] Valadi H., Ekstrom K., Bossios A., Sjostrand M., Lee J.J., Lotvall J.O. (2007). Exosome-mediated transfer of mRNAs and microRNAs is a novel mechanism of genetic exchange between cells. Nat. Cell Biol..

[4] Sung B.H., Weaver A.M. (2017). Exosome secretion promotes chemotaxis of cancer cells. Cell Adhes. Migrat..

[5] Hoshino A., Costa-Silva B., Shen T.L., Rodrigues G., Hashimoto A., Tesic Mark M., Molina H., Kohsaka S., Di Giannatale A., Ceder S., Singh S., Williams C., Soplop N., Uryu K., Pharmer L., King T., Bojmar L., Davies A.E., Ararso Y., Zhang T., Zhang H., Hernandez J., Weiss J.M., Dumont-Cole V.D., Kramer K., Wexler L.H., Narendran A., Schwartz G.K., Healey J.H., Sandstrom P., Labori K.J., Kure E.H., Grandgenett P.M., Hollingsworth M.A., de Sousa M., Kaur S., Jain M., Mallya K., Batra S.K., Jarnagin W.R., Brady M.S., Fodstad O., Muller V., Pantel K., Minn A.J., Bissell M.J., Garcia B.A., Kang Y., Rajasekhar V.K., Ghajar C.M., Matei I., Peinado H., Bromberg J., Lyden D. (2015). Tumour exosome integrins determine organotropic metastasis. Nature.

[6] Park E.J., Prajuabjinda O., Soe Z.Y., Darkwah S., Appiah M.G., Kawamoto E., Momose F., Shiku H., Shimaoka M. (2019). Exosomal regulation of lymphocyte homing to the gut. Blood Adv.

[7] Wang J., Hendrix A., Hernot S., Lemaire M., De Bruyne E., Van Valckenborgh E., Lahoutte T., De Wever O., Vanderkerken K., Menu E. (2014). Bone marrow stromal cell-derived exosomes as communicators in drug resistance in multiple myeloma cells. Blood.

[8] Wei G., Jie Y., Haibo L., Chaoneng W., Dong H., Jianbing Z., Junjie G., Leilei M., Hongtao S., Yunzeng Z., Junbo G. (2017). Dendritic cells derived exosomes migration to spleen and induction of inflammation are regulated by CCR7. Sci. Rep..

[9] Shen B., Liu J., Zhang F., Wang Y., Qin Y., Zhou Z., Qiu J., Fan Y. (2016). CCR2 positive exosome released by mesenchymal stem cells suppresses macrophage functions and alleviates ischemia/reperfusion-induced renal injury. Stem Cell. Int..

[10] Yang H.C., Zhang M., Wu R., Zheng H.Q., Zhang L.Y., Luo J., Li L.L., Hu X.Q. (2020). C-C chemokine receptor type 2-overexpressing exosomes alleviated experimental post-stroke cognitive impairment by enhancing microglia/macrophage M2 polarization. World J. Stem Cell..

[11] Liang Y., Qiao L., Peng X., Cui Z., Yin Y., Liao H., Jiang M., Li L. (2018). The chemokine receptor CCR1 is identified in mast cell-derived exosomes. Am J Transl Res.

[12] Marchese A. (2014). Endocytic trafficking of chemokine receptors. Curr. Opin. Cell Biol..

[13] Stone M.J., Hayward J.A., Huang C., Z E.H., Sanchez J. (2017). Mechanisms of regulation of the chemokine-receptor network. Int. J. Mol. Sci..

[14] Marchese A., Chen C., Kim Y.M., Benovic J.L. (2003). The ins and outs of G protein-coupled receptor trafficking. Trends Biochem. Sci..

[15] Canals M., Scholten D.J., de Munnik S., Han M.K., Smit M.J., Leurs R. (2012). Ubiquitination of CXCR7 controls receptor trafficking. PLoS One.

[16] Mueller A., Kelly E., Strange P.G. (2002). Pathways for internalization and recycling of the chemokine receptor CCR5. Blood.

[17] Mackie D.I., Nielsen N.R., Harris M., Singh S., Davis R.B., Dy D., Ladds G., Caron K.M. (2019). RAMP3 determines rapid recycling of atypical chemokine receptor-3 for guided angiogenesis. Proc. Natl. Acad. Sci. U. S. A.

[18] Imai T., Hieshima K., Haskell C., Baba M., Nagira M., Nishimura M., Kakizaki M., Takagi S., Nomiyama H., Schall T.J., Yoshie O. (1997). Identification and molecular characterization of fractalkine receptor CX_3_CR1, which mediates both leukocyte migration and adhesion. Cell.

[19] Nishimura M., Umehara H., Nakayama T., Yoneda O., Hieshima K., Kakizaki M., Dohmae N., Yoshie O., Imai T. (2002). Dual functions of fractalkine/CX3C ligand 1 in trafficking of perforin+/granzyme B+ cytotoxic effector lymphocytes that are defined by CX3CR1 expression. J. Immunol..

[20] Geissmann F., Jung S., Littman D.R. (2003). Blood monocytes consist of two principal subsets with distinct migratory properties. Immunity.

[21] Staumont-Salle D., Fleury S., Lazzari A., Molendi-Coste O., Hornez N., Lavogiez C., Kanda A., Wartelle J., Fries A., Pennino D., Mionnet C., Prawitt J., Bouchaert E., Delaporte E., Glaichenhaus N., Staels B., Julia V., Dombrowicz D. (2014). CX(3)CL1 (fractalkine) and its receptor CX(3)CR1 regulate atopic dermatitis by controlling effector T cell retention in inflamed skin. J. Exp. Med..

[22] Furio E., Garcia-Fuster M.J., Redon J., Marques P., Ortega R., Sanz M.J., Piqueras L. (2018). CX3CR1/CX3CL1 Axis mediates platelet-leukocyte adhesion to arterial endothelium in younger patients with a history of idiopathic deep vein thrombosis. Thromb. Haemostasis.

[23] Erreni M., Siddiqui I., Marelli G., Grizzi F., Bianchi P., Morone D., Marchesi F., Celesti G., Pesce S., Doni A., Rumio C., Roncalli M.G., Laghi L., Mantovani A., Allavena P. (2016). The fractalkine-receptor Axis improves human colorectal cancer prognosis by limiting tumor metastatic dissemination. J. Immunol..

[24] Conroy M.J., Lysaght J. (2020). CX3CL1 signaling in the tumor microenvironment. Adv. Exp. Med. Biol..

[25] Johnson L.A., Jackson D.G. (2013). The chemokine CX3CL1 promotes trafficking of dendritic cells through inflamed lymphatics. J. Cell Sci..

[26] Brown M., Johnson L.A., Leone D.A., Majek P., Vaahtomeri K., Senfter D., Bukosza N., Schachner H., Asfour G., Langer B., Hauschild R., Parapatics K., Hong Y.K., Bennett K.L., Kain R., Detmar M., Sixt M., Jackson D.G., Kerjaschki D. (2018). Lymphatic exosomes promote dendritic cell migration along guidance cues. J. Cell Biol..

[27] Thery C., Amigorena S., Raposo G., Clayton A. (2006). Isolation and characterization of exosomes from cell culture supernatants and biological fluids. Curr. Protoc. Cell Biol..

[28] Soe Z.Y., Prajuabjinda O., Myint P.K., Gaowa A., Kawamoto E., Park E.J., Shimaoka M. (2019). Talin-2 regulates integrin functions in exosomes. Biochem. Biophys. Res. Commun..

[29] Conroy M.J., Maher S.G., Melo A.M., Doyle S.L., Foley E., Reynolds J.V., Long A., Lysaght J. (2018). Identifying a novel role for fractalkine (CX3CL1) in memory CD8(+) T cell accumulation in the omentum of obesity-associated cancer patients. Front. Immunol..

[30] Fogg D.K., Sibon C., Miled C., Jung S., Aucouturier P., Littman D.R., Cumano A., Geissmann F. (2006). A clonogenic bone marrow progenitor specific for macrophages and dendritic cells. Science.

[31] Bain C.C., Mowat A.M. (2011). Intestinal macrophages - specialised adaptation to a unique environment. Eur. J. Immunol..

[32] Piper R.C., Katzmann D.J. (2007). Biogenesis and function of multivesicular bodies. Annu. Rev. Cell Dev. Biol..

[33] Reggiori F., Pelham H.R. (2001). Sorting of proteins into multivesicular bodies: ubiquitin-dependent and -independent targeting. EMBO J..

[34] Hessvik N.P., Llorente A. (2018). Current knowledge on exosome biogenesis and release. Cell. Mol. Life Sci..

[35] Wei Z., Batagov A.O., Carter D.R., Krichevsky A.M. (2016). Fetal bovine serum RNA interferes with the cell culture derived extracellular RNA. Sci. Rep..

[36] Shelke G.V., Lasser C., Gho Y.S., Lotvall J. (2014). Importance of exosome depletion protocols to eliminate functional and RNA-containing extracellular vesicles from fetal bovine serum. J. Extracell. Vesicles.

