# Supplementary material for: Ligand-competent fractalkine receptor is expressed on exosomes
Source: Biochem Biophys Rep. 2021 Feb 2;26:100932. doi: 10.1016/j.bbrep.2021.100932 (PMC7859287; doi:10.1016/j.bbrep.2021.100932)
Supplement: Multimedia component 1 [file mmc1.pdf]

Supplemental Figure 1

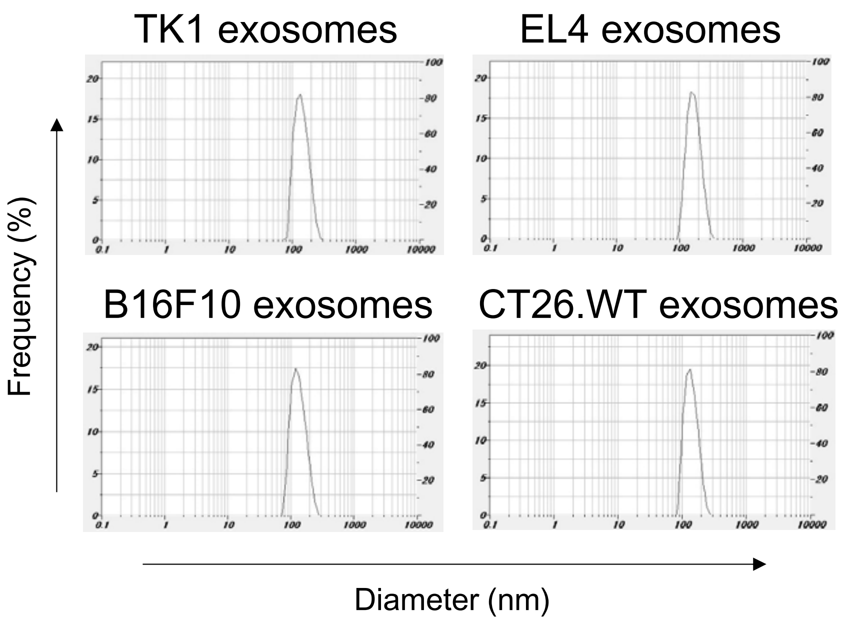

Supplemental Figure 1. Dynamic light scattering (DLS) analysis. The diameters were measured with the exosomes isolated from several selected cell lines including TK1, EL4, B16F10, and CT26.WT. The data are the representative of 2-3 independent experiments.

Supplemental Figure 2

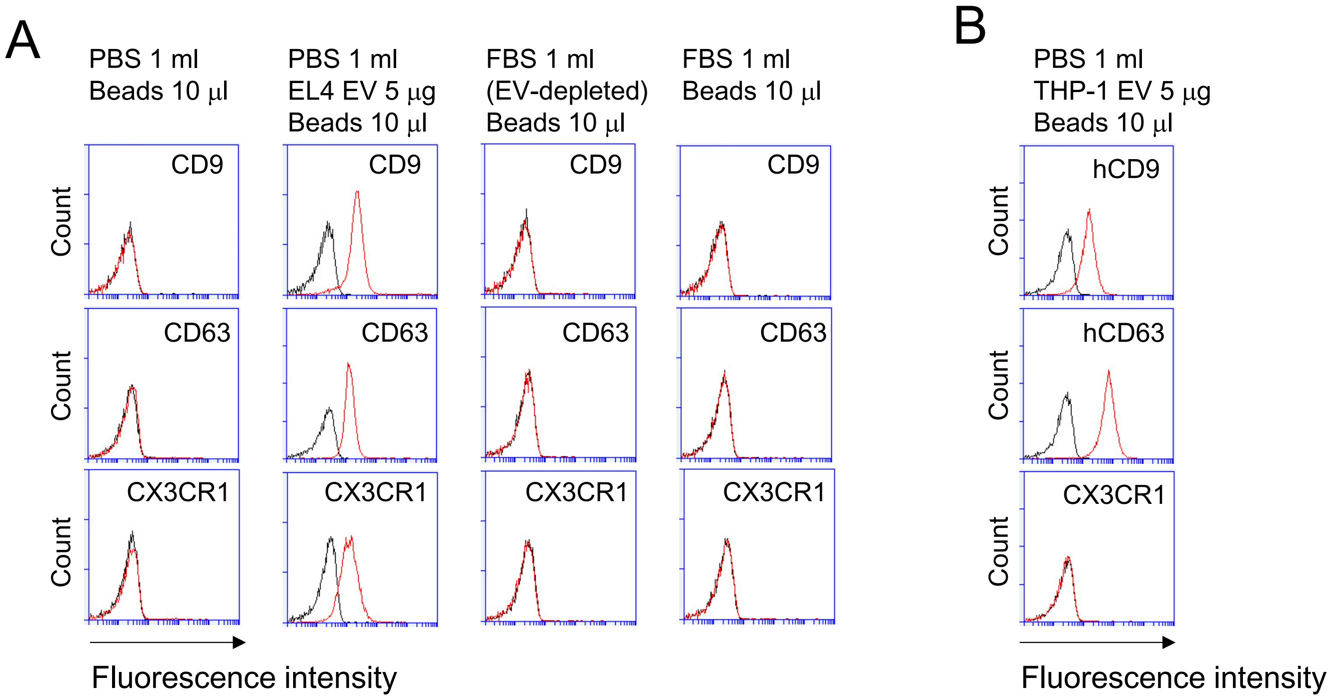

Supplemental Figure 2. Analysis of CX3CR1 and tetraspanin (CD9 and CD63) expressions on the samples incubated with those indicated. (**A**) To test if the possibly remained EVs in exosome-depleted FBS affect the exosomal expression of CX3CR1 as well as tetraspanins, the samples were conjugated with 10 μl beads as done with the same methods as exosomes and analyzed their expressions in flow cytometry. (**B**) The EVs (exosomes) of THP-1 (human monocytes) conjugated with beads and analyzed in flow cytometry, which were positive for human CD9 and CD63, didn't show any CX3CR1 expression by the antibody used in this study. Representative histograms show the expression of indicated markers. Data are representative of three separate analyses. All monoclonal antibodies (MAb) indicated were used for anti-mouse MAb, except for human CD9 (hCD9) and hCD63. Red lines, MAb; and black lines, isotype.
